# In Silico and In Vivo Evaluation of SARS-CoV-2 Predicted Epitopes-Based Candidate Vaccine

**DOI:** 10.3390/molecules26206182

**Published:** 2021-10-13

**Authors:** Mahmoud M. Shehata, Sara H. Mahmoud, Mohammad Tarek, Ahmed A. Al-Karmalawy, Amal Mahmoud, Ahmed Mostafa, Mahmoud M. Elhefnawi, Mohamed A. Ali

**Affiliations:** 1 Center of Scientific Excellence for Influenza Viruses, National Research Centre, Giza 12622, Egypt; sarahussein9@yahoo.com (S.H.M.); ahmed_elsayed@daad-alumni.de (A.M.); mohamedahmedali2004@yahoo.com (M.A.A.); 2 Institute of Medical Virology, Justus Liebig University Giessen, 35392 Giessen, Germany; 3 Bioinformatics Department, Armed Forces College of Medicine (AFCM), Cairo 11757, Egypt; Motarek_022_3@afcm.edu.eg; 4 Department of Pharmaceutical Medicinal Chemistry, Faculty of Pharmacy, Horus University-Egypt, New Damietta 34518, Egypt; akarmalawy@horus.edu.eg; 5 Department of Biology, College of Science, Imam Abdulrahman Bin Faisal University, P.O. Box. 1982, Dammam 31441, Saudi Arabia; 6 National Research Centre, Biomedical Informatics and Cheminformatics Group, Informatics and Systems Department, Cairo 12622, Egypt; mahef111@gmail.com

**Keywords:** SARS-CoV-2, prediction, epitopes, vaccine

## Abstract

Severe Acute Respiratory Syndrome Coronavirus 2 (SARS-CoV-2, the causative agent of coronavirus disease (COVID-19)) has caused relatively high mortality rates in humans throughout the world since its first detection in late December 2019, leading to the most devastating pandemic of the current century. Consequently, SARS-CoV-2 therapeutic interventions have received high priority from public health authorities. Despite increased COVID-19 infections, a vaccine or therapy to cover all the population is not yet available. Herein, immunoinformatics and custommune tools were used to identify B and T-cells epitopes from the available SARS-CoV-2 sequences spike (S) protein. In the in silico predictions, six B cell epitopes QTGKIADYNYK, TEIYQASTPCNGVEG, LQSYGFQPT, IRGDEVRQIAPGQTGKIADYNYKLPD, FSQILPDPSKPSKRS and PFAMQMAYRFNG were cross-reacted with MHC-I and MHC-II T-cells binding epitopes and selected for vaccination in experimental animals for evaluation as candidate vaccine(s) due to their high antigenic matching and conserved score. The selected six peptides were used individually or in combinations to immunize female Balb/c mice. The immunized mice raised reactive antibodies against SARS-CoV-2 in two different short peptides located in receptor binding domain and S2 region. In combination groups, an additive effect was demonstrated in-comparison with single peptide immunized mice. This study provides novel epitope-based peptide vaccine candidates against SARS-CoV-2.

## 1. Introduction

A novel coronavirus strain called SARS-CoV-2 was discovered in Wuhan, China in December 2019 [1]. Since then there have been 216 million cases with coronavirus disease 2019 (COVID-19) including almost 4.5 million deaths globally according to WHO records [2]. SARS-CoV-2 is single strand positive sense RNA genome with a molecular size around 30,000 bp in length [3] and classified as belonging to the *Nidovirales* order, *Coronaviridae* family. There are four different genera in the *Coronaviridae* family; alpha, beta, gamma and delta groups. The alpha and beta clusters include seven human coronaviruses (CoVs) strains like HKU1, 229E, OC43, SARS-CoV, MERS-CoV and SARS-CoV-2 [3]. Bats are suspected as being the origin of most CoVs [4].

Recently, several variants were generated and characterized like Alpha (United Kingdom-B.1.1.7), Beta (South Africa-B.1.351), Gamma (Brazil-P.1) and Delta (India-B.1.617.2) which display increased infection transmission rates [2]. The structural proteins of SARS-CoV-2 are S, envelope (E), membrane (M), nucleocapsid (N) among which S protein contains the major structural immunogenic epitopes that induce the immune system response [5].

The receptor-binding domain (RBD) bonded with angiotensin-converting enzyme 2 (ACE2) cellular receptor which describes the structural bases for viral infection depends on S protein [6]. The stimulation of neutralizing antibodies (nAb) and ACE2 binding step in viral entry are intermediated by RBD of the S protein [7].

Until now, there are seven approved SARS-CoV-2 vaccines in phase 4 that were either finally approval or received emergency licensure by WHO; two inactivated whole virus vaccines (SinoVac (Beijing, China) and Sinopharm (Beijing, China)), three virus-like particle (VLP) expressing S protein (the AstraZeneca (Cambridge, UK), Johnson & Johnson (New Brunswick, NJ, USA) and CanSino Biological (Tianjin, China) ones) and two mRNA vaccines (Moderna (Cambridge, MA, USA) and Pfizer (New York, NY, USA)/BioNTech (Mainz, Germany)) [8]. By 5 October 2021, there were 124 candidate vaccines in clinical trials stages and 194 in pre-clinical development on different vaccine platforms like inactivated virus, subunit, VLP, mRNA, live attenuated virus, DNA, bacterial antigen-spore expression vector and protein subunit vaccines [8].

Some in silico studies were implemented to predict immunogenic epitopes in S protein targeting T-cells and B-cells binding recognition for production if specific antibodies against SARS-CoV-2 [9,10,11]. The bioinformatics prediction analysis for MERS-CoV, SARS-CoV and SARS-CoV-2 revealed a conserved epitope binding to B and T cells between SARS-CoV and SARS-CoV-2 due to high genetic similarity between both viruses [12].

Certain regions of SARS-CoV and SARS-CoV-2 receptor binding domain (RBD) could provide strong potential vaccine targets for a more specialized immune response against binding-critical portions of the RBD [5]. These binding and cleavage-essential segments have been previously identified as regions covering continuous sequences on the spike glycoprotein including RBDp (455–502), RBDg (397–437), furin cleavage site in the S1/S2 (676–690) and TMPRSS2 cleavage site in the S2 region (802–816) [13,14].

Until now, no in vivo data have been published for the SARS-CoV-2 predicted peptides as a vaccine candidate. Herein, prediction tools and an analysis of S protein of SARS-CoV-2 were implemented and the predicted immunogenic peptide epitopes were synthesizes and inoculated in vivo on female Balb/c mice for evaluation as a vaccine candidate.

## 2. Results

### 2.1. Computational Design of Neutralizing Epitopes

Linear neutralizing epitopes were predicted using the Bepipred-2.0 algorithm (Table 1). Six peptides scored positively above the average (Score > 0.2) and were subsequently identified as potential linear neutralizing epitopes. These peptides were named alphabetically (A–D, Table 1). IFN-γ inducing epitopes were also predicted using the IFNepitope tool and were sorted according to scores. Negative scores indicating negative predictions were excluded (Table 1). Epitopes that did not cover any of the studied variants were also excluded.

### 2.2. Structural Configuration of Spike ACE2 Interaction

**Figure 1:**
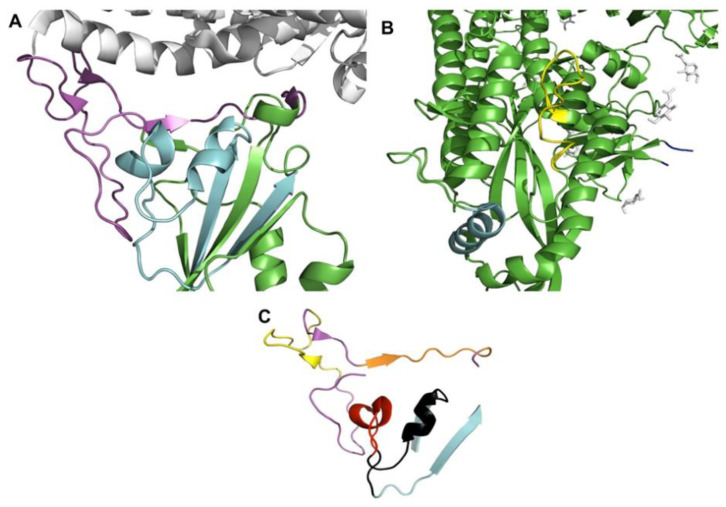
(**A**) Shows the structural configuration of the spike glycoprotein (green) binding with receptor ACE2 (white) (PDB: 7DF4). The receptor-binding domain of the spike glycoprotein shows two distinctive regions essential for binding; RBDg (cyan) and RBDp (magenta). (**B**) Shows the cleavage sites of TMPRSS2 (yellow), furin (blue) in addition to N-acetylglucosamine (NAG) moieties (white). (**C**) shows the selected epitopes within RBDg (peptides A, D) and RBDp (peptides B, C) colored differently including: peptide A (red), peptide B (yellow), peptide C (black), peptide D (orange).

Identification of specific regions within RBD that provides the binding interface was previously identified through structural analysis of the interaction between RBD and ACE2. RBD-ACE2 interaction has speculated to be established regardless of the bound/unbound state of ACE2. In addition, RBD was founded to interact with ACE2 glycosylated interface in a pattern that could explain the antiviral effect of some compounds against SARS-CoV-2. In addition, two S-glycoprotein residues namely Gly416 and Lys417 were computationally identified to be responsible for the interaction with the N-acetylglucosamine (NAG) moiety, this observation had led to identification of another binding critical region within SARS-CoV-2 RBD [13]. These binding-essential and cleavage-essential segments have been identified as regions covering continuous sequences on the spike glycoprotein including; RBDg (397–437), RBDp (455–502) and TMPRSS2 Cleavage site in the S2 (802–816). The receptor-binding domain of the spike glycoprotein shows 2 distinctive regions essential for binding with ACE2 receptor which four predicted epitopes (A–D) were found on these sites as illustrated in Figure 1.

### 2.3. Predicted Epitopes and VOCs

**Figure 2:**
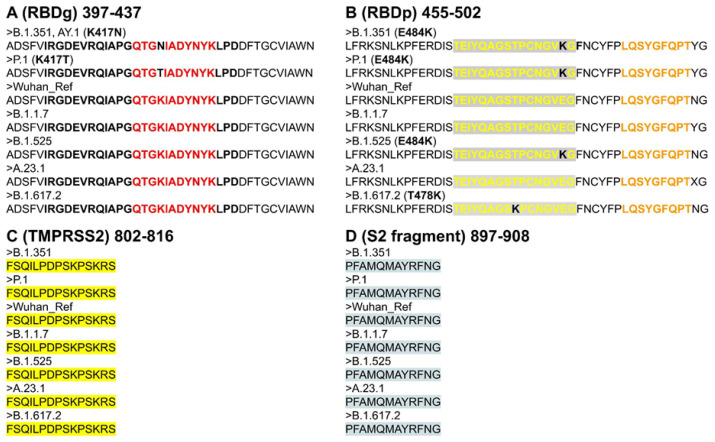
(**A**) Shows the amino-acid sequence alignment of RBDg epitopes among SARS-CoV-2 variants of concern (VOCs). Peptide A (bold red) is conserved in five variants out of seven, however it harbors K417N mutation within variants B.1.351 and B.1.617.2.1, In-addition to mutation K417T within P.1 variant. Peptide D (bold black) also includes the aforementioned mutations. (**B**) Shows the amino-acid sequence alignment of RBDp epitopes among SARS-CoV-2 VOCs. While peptide C (bold orange) is conserved among all studied variants, peptide B (bold yellow) is only conserved among three variants including; Wuhan reference, B.1.1.7 and A.23.1. In addition, peptide B harbors mutation E484K within B.1.351, B.1.525 and P.1 variants, peptide B also includes a delta variant B.1.617.2 T478K mutation. (**C**) Shows peptide E targeting TMPRSS2 cleavage site (yellow highlight) is completely conserved within the studied variants. (**D**) Shows peptide F targeting S2 fragment (cyan highlight) is completely conserved within the studied variants.

**Figure 3:**
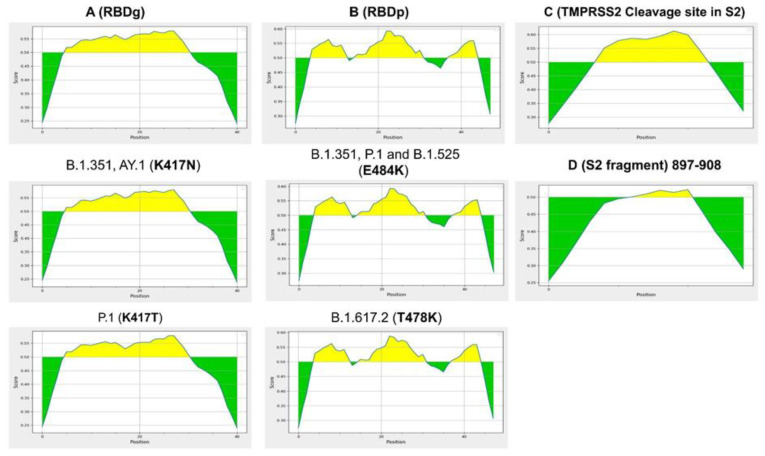
Shows the Bepipred 2.0 predictions for neutralizing antibody epitopes within RBD and cleavage regions of interest. The figure also shows the effect of evolved mutations of different SARS-CoV-2 variants on epitope prediction. (**A**) Shows that mutation K417N of variants B.1.351 and AY.1 does not affect the prediction score for RBDg epitopes A and D. However, mutation K417T of variant P.1 causes mild decrease in prediction scores which still does not affect the positivity of the selected epitopes. (**B**) Shows that neither E484K nor T478K negatively affect the positivity of RBDp peptides B and C. (**C**) Shows the positivity of peptide E Bepipred 2.0 prediction within the TMPRSS2 cleavage site region. (**D**) Shows the positivity of peptide F within S2 fragment (897–908).

The six epitopes scored positively within 15-kmers for potential induction of IFN-γ response. Epitopes C (RBDg) and E (TMPRSS2 cleavage site) were found to virtually cover all SARS-CoV-2 VOCs (Table 1, Figure 2). Epitope B (RBDg) was predicted to induce IFN-γ. Moreover, Both RBDp epitopes A and D were found to cover A.1, B.1.525, B.1.1.7 and A.23.1 variants. Peptide E was conserved between all VOCs, which located post fusion peptide region at S2 fragment. Epitope A and D are conserved within Wuhan reference, B.1.1.7, A.23.1, B.1.525 and B.1.617.2, however it harbors K417N and K417T mutations within variants B.1.351 and P.1 respectively. Epitope B is only conserved among three variants, including Wuhan reference, B.1.1.7 and A.23.1. In addition, Epitope B harbors mutation E484K within B.1.351, B.1.525 and P.1 variants, peptide B also includes a delta variant T478K mutation (Figure 3).

These variants could possibly alter the antibody response generated by the selected epitopes, thus, Bepipred predictions for each region of interest were repeated upon mutating of each region with the variant of interest as shown in Figure 3. Interestingly, none of the studied mutations, including K417N, K417T, T478K and E484K, were able to negatively affect the positivity of neutralization prediction for each of the five selected peptides using Bepipred 2.0 (Figure 3). However, mutation K417T of variant P.1 was found to cause a mild decrease in the positive predictions of RBDg epitopes. This finding is in line with clinical trials showing the effectiveness of vaccines designed on the basis of the original Wuhan reference sequence of SARS-CoV-2 to elicit sufficient neutralizing antibody responses against circulating evolved variants of SARS-CoV-2 [15].

### 2.4. Mice Experiment

**Figure 4:**
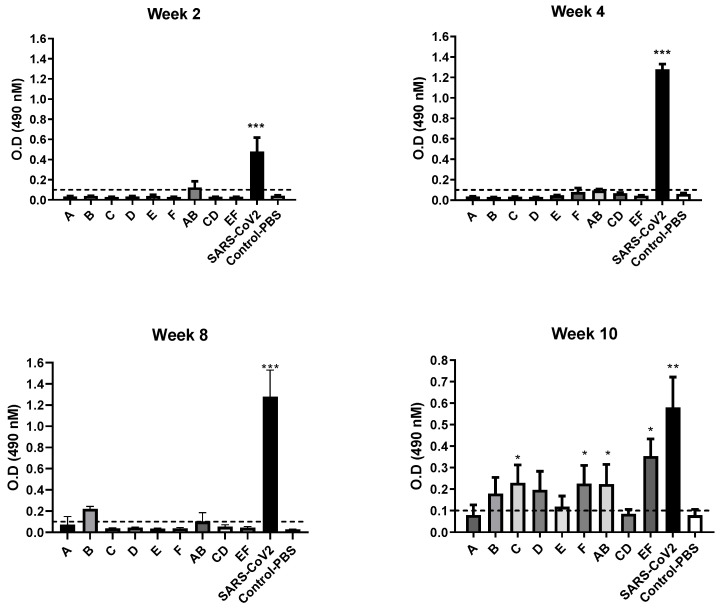
Optical density (O.D) obtained from ELISA for sera of BALB/C mice immunized with short-peptides from A to E and combination (AB, CD, EF) groups in comparison with SARS-CoV-2 inactivated vaccine. Cut-off value for assay validation = 0.1 O.D reading at 490 nM. Statistical changes marked by * *p* value < 0.05, ** *p* value < 0.01 and *** *p* value < 0.001.

The immunized female BALB/c mice produced a significant neutralizing antibody (nAb) against SARS-CoV-2 inactivated vaccine for groups injected with C and F short peptides with doses 10 µg in comparison with control (PBS) group. The elicited antibody response of mice as illustrated in Figure 4 showed a highly significant (*p* value < 0.001) increase in O.D reading of SARS-CoV-2 inactivated group in comparison with control group and peptides groups at weeks 2, 4, 8 and 10 post-vaccination. In AB and EF combination vaccinated mice groups showed a significant increase in O.D reading at week 10 post-vaccination (*p* value < 0.001) and a significant increment of antibody response against individual vaccinated groups at weeks 10 post vaccination. This means that the combination of these AB and EF groups had an additive effect for producing high nAb compared with single peptide vaccination.

## 3. Discussion

More than 140 candidate vaccines against coronavirus have been evaluated in order to deliver safe and effective vaccines worldwide against the COVID-19 pandemic [16]. Scientists all over the globe battle to find safe and effective candidate vaccines.

Protein S is a major therapeutic target for producing nAb the most elicited from SARS-CoV-2 infection in-which S contains immunogenic epitopes and RBD on its surface. So, researchers used S protein in vaccination strategies against pandemic COVID-19 disease [5,9]. These immunogenic S epitopes were bounded to CD4 and CD8 inducing T cells to present MHC II and stimulate cellular immunity [9].

Immune-informatics is playing a significant role in vaccine development and immunogenic data analysis against different viruses. The specificity of epitope vaccines was enhanced by selecting the antigenic surface epitopes that elicit potent immunity [17,18]. The S protein plays a crucial role in the entry and attachment of viral particles with host cell receptor and high antigenic surface protein [6,7].

The current study was implemented to predict and evaluate these peptides as a safe approach for vaccination against SARS-CoV-2. Both B and T cells of the adaptive immune response were selected in our prediction model for getting best matching immunogenic epitopes as candidate vaccines Table 1. These peptides are IFN-γ inducing predicted epitopes and cross-react with B cells inducing humoral immune response. Four peptides (A–D) were located in S1 region and located inside RBD and two peptides (E, F) located at S2 region the more conserved part of S protein as shown in Figure 1. The selected peptides were positive in neutralizing prediction using Bepipred 2.0, although the new VOCs mutations found in peptides A and B in some variants (Figure 3).

The four remaining peptides (C–F) are conserved for all VOCS. Other researchers predicted the same peptide sequence PFAMQMAYRFNG (peptide F) and recommended it as SARS-CoV candidate vaccine [19], as it was conserved between both SARS-CoV and SARS-CoV-2. In addition, peptide QTGKIADYNYK (E) is predicted by other authors to bind and induce HLA alleles using NetMHCpan (I and II) and MHC flurry database [20].

A lot of in silico peptide-based prediction studies were implemented to establish a candidate vaccines based on binding with B and T cells with another programs and databases [9,21,22,23] which lack in vitro or in vivo verification studies.

Therefore, to evaluate the immunoinformatics predicted peptides for elucidation of nAb, these peptides were injected intra-muscular individually and in three combinations randomly in Balb/c mice. The collected sera from mice were evaluated by ELISA to measure nAb. The results showed a significant increase of nAb in two combinations (AB and EF) and also for the individual C and F peptides compared to unvaccinated mice as presented in Figure 4. These results confirm that the prediction model and analysis in our study is running in a good manner and extra work for cellular immunity evaluation is needed. The immunoinformatics approaches showed that vaccine-based epitopes designed against SARS-CoV-2 conceded promising results by inducing an immune response in vivo. Therefore, related to our data, an extra animal experiment and clinical study are highly recommended for these designed immunogenic candidate peptides vaccines for studying their efficacy, safety and immunogenic parameters as new candidate epitope-based vaccines.

## 4. Materials and Methods

### 4.1. Analysis of the Receptor Binding Domain for Epitope Prediction

Structural annotation of these portions was done using PyMol (version 2.5.1, DeLano Scientific LLC, South San Francisco, CA, USA) based on a structurally solved model of spike glycoprotein in complex with ACE2 receptor [24], the model is deposited at the Protein Bank Database under PDB ID (7DF4) [25].

Then, Bepipred-2.0 (http://tools.iedb.org/bcell/, accessed on 1 August 2021) was used to predict linear neutralizing epitopes within the binding and cleavage-essential portions of the RBD [26]. Then, the IFN epitope tool (https://crdd.osdd.net/raghava/ifnepitope/, accessed on 1 August 2021) was also used to predict IFN-γ inducing k-mer epitopes [27]. To study the associations between evolved variants of SARS-CoV-2 and our selected epitopes, sequences of spike glycoprotein from SARS-CoV-2 variants of concern (VOCs) were retrieved from the GISAID database including; Wuhan Reference variant, P.1, B.1.1.7, B.1.351, B.1.617.2 and two of the noted variants A.23.1 and B.1.525. Subsequently, associated mutations in each variant were mapped to their corresponding locations on the amino acid sequences of our regions of interest.

### 4.2. Synthesis of the Peptides Panel

The designated short peptides were synthesized with a cysteine residue at the C-terminal by (ABI Scientific, Sterling, VA, USA) and dissolved in dimethyl sulfoxide at concentration 2 mg/mL. Besides, the peptides were been conjugated at the N-terminus with keyhole-limpet-haemocyanin (KLH) [28,29]. The synthesized peptides were been analyzed by HPLC and mass spectroscopy to confirm their identity.

### 4.3. Preparation of Inactivated SARS-CoV-2 Antigen

SARS-CoV-2 isolate (hCoV-19/Egypt/NRC-03/2020) with accession number EPI_ISL_430820 (available at https://www.gisaid.org/, accessed on 1 August 2021) was propagated at Vero-E6 cells (ATCC, CRL-1586) in T-175 flask with DMEM media containing 1% fetal bovine serum, 100 U/mL penicillin and 100 μg/mL streptomycin (GIBCO) at 37 °C in 5% CO_2_. The cell culture supernatant after 5 days post infection was been harvested and centrifuged at 1800× *g* for 10 min to remove cell debris.

Inactivation of cell culture clarified harvest using β-propiolactone 0.1% *v*/*v* in a shaking incubator for 48 h at 4 °C. A total 15 mL inactivated batch mixed with 6 mL sucrose 20% then ultracentrifuged at 50,000 rpm/min for 1 h at 4 °C (Sorvall MTX 150, Thermo Fisher Scientific, Waltham, MA, USA) then eluted in 1 mL 1× PBS. The protein content was measured using PierceTM BCA Protein Assay kit (Thermo) following the manufacturer’s instructions.

### 4.4. Animal Experiment

**Figure 5:**
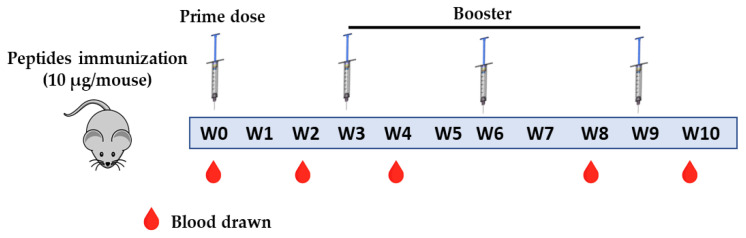
Timeline of the immunization protocol of predicted short peptides in BALB/C mice. W; week.

The 6–8 weeks female BALB/C mice were housed in a specified pathogen-free unit at National Research Centre (NRC), Egypt with water and food *ad libitum*, according to NRC guidelines of animal care and use committee. Mice were housed under 12/12 h light/dark cycles at room temperature ranged from 22–26 °C and relative humidity from 30–60%. Mice were immunized with the six predicted short peptides listed in Table 1 individually (7 mice/group) and 3 combinations between synthesized peptides AB, CD and EF. The intramuscular immunization was implemented with 10 µg/mice dose [30], mixed with alum adjuvant (Thermo) by ratio (1:4) alum:antigen. The animals received a prime dose and three boosters at a three week interval as shown in Figure 5. The inactivated SARS-CoV-2 group was immunized intramuscular with prime and one booster dose of 6 µg/mice according to Kandeil et al. [31]. Blood was collected from sinusoidal orbital vein from mice at different time points at 0, 2, 4, 8, 10 weeks post-vaccination.

### 4.5. ELISA Assay

The collected sera at different time points were heat inactivated at 56 °C for 1 h in water bath then stored at 4 °C. ELISA was performed according to Kandeil et al. [31]. Briefly, 96 well ELISA plates were coated with 50 µL per well 1× coating buffer (SeraCare, Milford, MA, USA) containing 20 µg/mL inactivated antigen then plates were incubated overnight at 4 °C. Plates were washed four times with 200 µL/well phosphate buffered saline, 0.1% tween 20 (T-PBS) then dried well. Two hundred µL per well of 5% non-fat milk powder (Santa Cruz Dallas, TX, USA) in T-PBS were added and incubated for 3 h at room temperature (RT). After blocking time, plates were washed four times with T-PBS then dried well. Sera were diluted in another 96 well plate in triplicate at 1:50 dilution for each serum sample in 200 µL 1% non-fat milk in T-PBS including positive and negative control sera. 50 µL/well of diluted sera samples were transferred to ELISA plates and incubated for 1.5 h at RT. After sera incubation at RT, plates were washed four times with T-PBS then dried. Secondary anti-Rat HRP-labeled (KBL) was diluted 1:3000 in 1% non-fat milk then 50 µL/well were added to all plates. After 1 h incubation at RT, plates were washed 4 times with T-PBS and dried. Substrate solution was prepared by dissolving *ortho*-phenylenediamine (OPD) tablets (ACROS Organics, Pittsburgh, PA, USA) in 20 mL phosphate-citrate buffer, a 50 µL/well substrate solution was added to all plates and incubated for 5 min until positive control sera were reacted then, 50 µL/well H_2_SO_4_ (3 M) stopping solution were added to stop the reaction. Plates were measured immediately after adding stopping solution in ELISA plate reader at 490 nM. Classen et al. [32], defined the cut-off value = twice or three times the mean absorbance obtained from the negative controls. So, a total of 15 negative control sera samples from Balb/c mice were measured by our in-house established ELISA assay, the mean ± SD absorbance reading = 0.0322 ± 0.006. Therefore, the three times value equal 0.0967 ± 0.018; the cut off value is ~0.1 O.D reading.

### 4.6. Statistical Analysis

The experiments were conducted in triplicates and the given data are presented as means ± SEM. Ordinary one-way ANOVA followed by Tukey multiple comparisons test with a single pooled variance was performed using GraphPad Prism version 9.1 (GraphPad Software, San Diego, CA, USA; www.graphpad.com, accessed on 1 August 2021).

## 5. Conclusions

In this study, the predicted epitopes showed B and T-cells with high conservancy, non-allergenicity, nontoxicity, high population coverage, and significant interaction with MHC class I and II alleles with a good affinity. The prediction data used in selecting candidate SARS-CoV-2 short peptides for conducting experimental vaccination of high immunogenic scores epitopes. Our results indicated a reliable promising induction of immune responses in mice after vaccination.

## Figures and Tables

**Table 1:** Bepipred 2.0 positive predictions (scoring > 0.5) for linear neutralizing epitopes covering targeted RBD and cleavage segments of the spike glycoprotein with targeted SARS-CoV-2 variants.

Short Peptide Names	Neutralizing Epitope Sequence	Location	SARS-CoV-2 Variants	IFNepitope Score
A	QTGKIADYNYK	RBDg *	Wuhan_Reference B.1.1.7, A.23.1 B.1.525, B.1.617.2	0.23869643
B	TEIYQAGSTPCNGVEG	RBDp *	Wuhan_Reference B.1.1.7, A.23.1	0.256374
C	LQSYGFQPT	RBDp *	Wuhan_Reference P.1, B.1.351, B.1.1.7 A.23.1, B.1.525, B.1.617.2	0.25635466
D	IRGDEVRQIAPGQTGKIADYNYKLPD	RBDg *	Wuhan_Reference B.1.1.7, A.23.1, B.1.525, B.1.617.2	0.22687041
E	FSQILPDPSKPSKRS	TMPRSS-2 Cleavage site in S2 *	Wuhan_Reference P.1, B.1.351, B.1.1.7 A.23.1, B.1.525, B.1.617.2	3.6814886
F	PFAMQMAYRFNG	S2 fragment	Wuhan_Reference P.1, B.1.351, B.1.1.7 A.23.1, B.1.525, B.1.617.2	3.5004815

* RBDg; spike amino acids (aa) sites 397–437, RBDp; aa 455–502 and S2; spike fragment 2.
